# Supplementary figures and images for: Mycobacterium tuberculosis complex drug-resistance, phylogenetics, and evolution in Nigeria: Comparison with Ghana and Cameroon
Source: PLoS Negl Trop Dis. 2023 Oct 12;17(10):e0011619. doi: 10.1371/journal.pntd.0011619 (PMC10597489; doi:10.1371/journal.pntd.0011619)

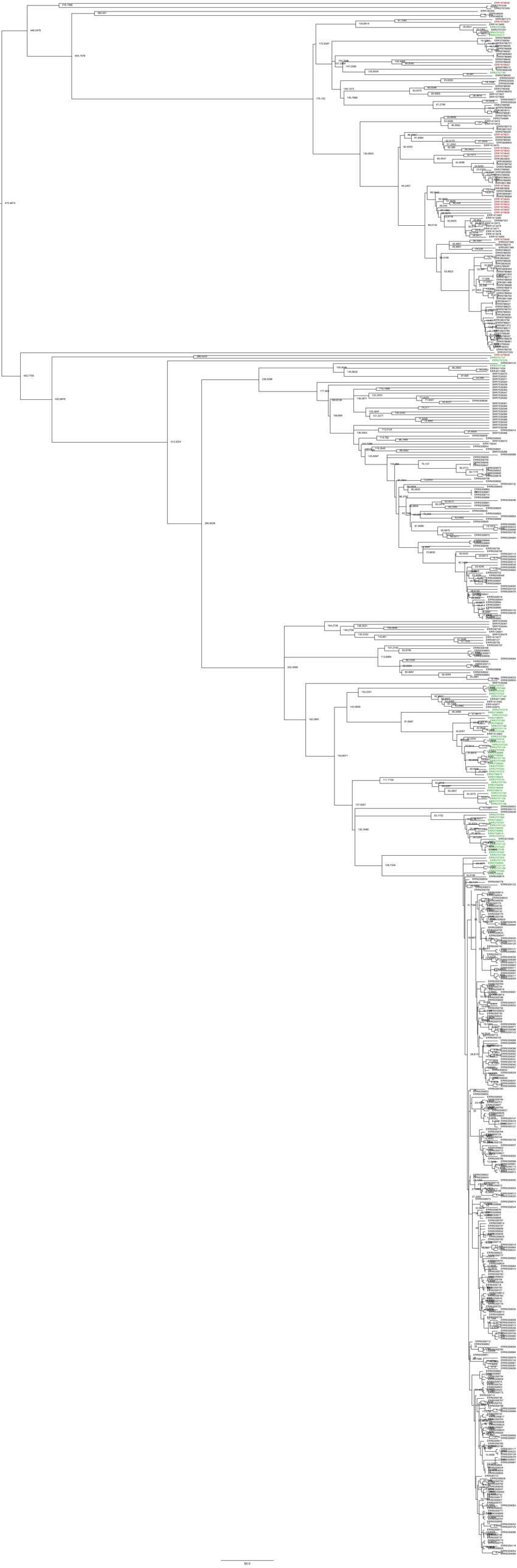

Supplement: S3 Material — (PDF) [file pntd.0011619.s003.pdf]

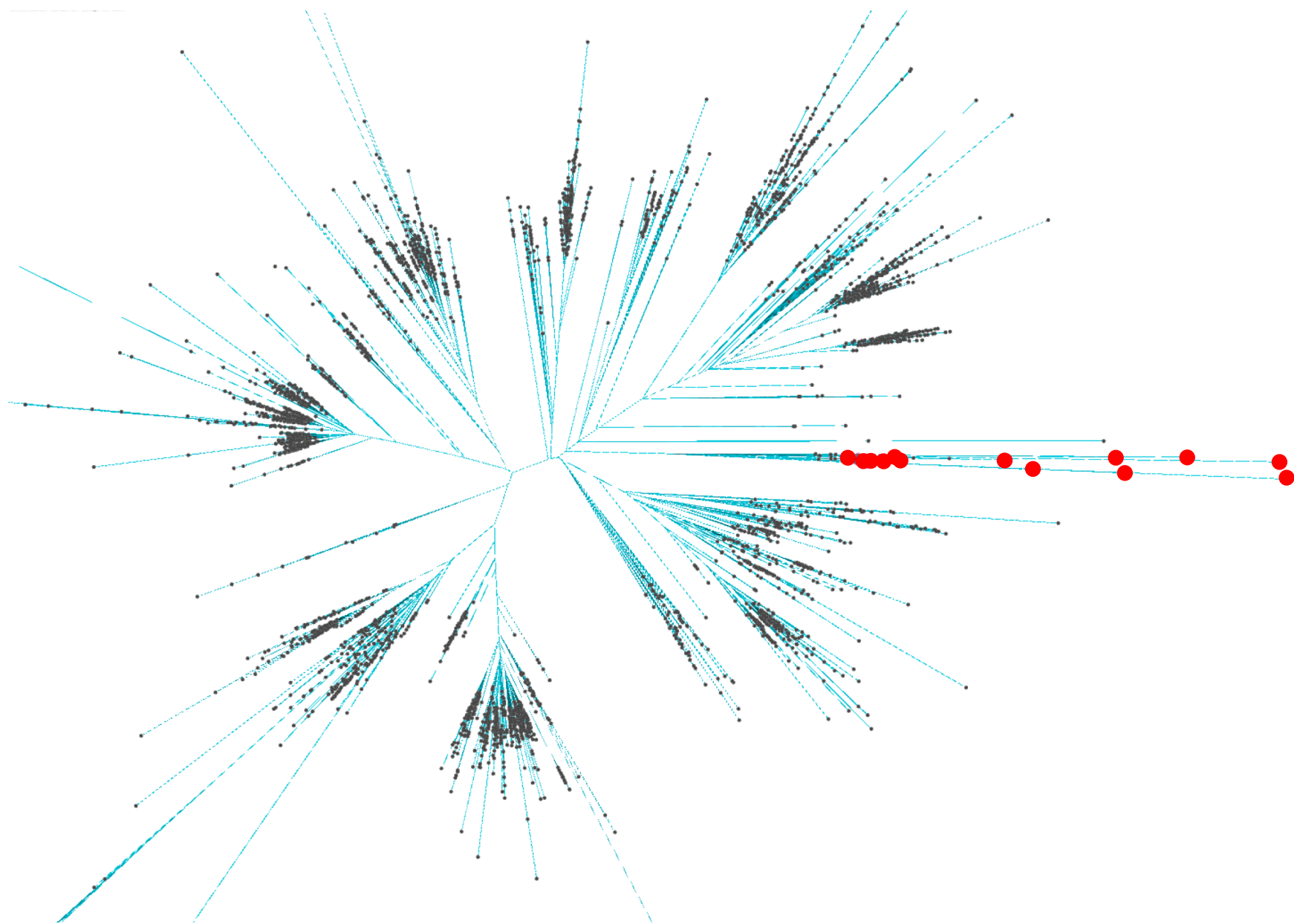

Supplement: S4 Material — (PDF) [file pntd.0011619.s004.pdf]

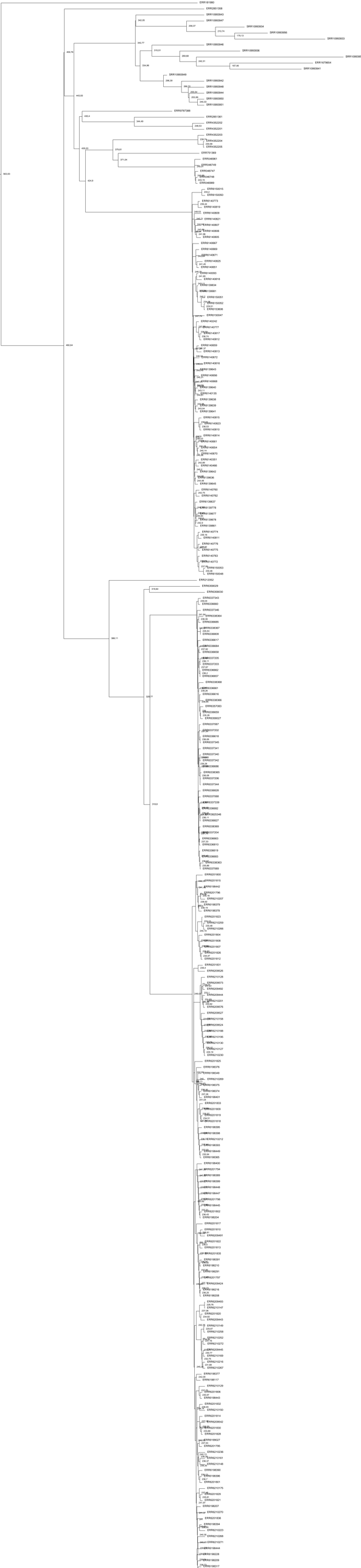

Supplement: S5 Material — The Nigerian clade is at the top of the tree. (PDF) [file pntd.0011619.s005.pdf]
