# Supplementary material for: Mycobacterium tuberculosis complex drug-resistance, phylogenetics, and evolution in Nigeria: Comparison with Ghana and Cameroon
Source: PLoS Negl Trop Dis. 2023 Oct 12;17(10):e0011619. doi: 10.1371/journal.pntd.0011619 (PMC10597489; doi:10.1371/journal.pntd.0011619)
Supplement: Attachment — Submitted filename: Answer_to_reviewers_PNTD_080823_MRS.doc [file pntd.0011619.s006.doc]

Reviewer #1: Please make clearer in which part(s) the 325 patients were used and in which the 165 patients

*"Concerning the new data, it consists on a set of 325 patients, among which 165 samples out of this patient cohort, were collected from TB regional lab in Aminu Kano Teaching Hospital [AKTH] from Northern States between 2019-2022, (Figure 1)*. "

The methods are appropriate for the objective, the link between the data and the conclusion must made clearer.

the text was changed as follows:

***Answer*** *: The cohort of 165 patients from the AKTH regional lab comprises of complete clinical histories from Nigerian states namely: Borno(5), Jigawa(5), Kano(98), Katsina(19), Kebbi (25) while 8 archive DNA of Nigerian origin both from Lawson et al. 2012 and Molina-Moya et al. 2018 which were obtained from the IGEPE lab, and 3 Isolates from Bichat Hopital (France) that where Included . The description is as follows.*

FR= France, NG= Nigeria

**The statistics used are not clearly stated.**

*Answer: We have now added a paragraph on statistics to describe how the PCA analysis was conducted in Section 1.6 of the Materials and Methods, which is now entitled "Statistics and Bioinformatics Analysis".*

the added paragraph is as found below:

*Principal component analysis was performed using the PCA function of the scikit-learn python library. The data matrix provided had as many rows as patients in the study, and as many columns as antibiotics. The data was standardised and slightly noisy (Gaussian noise, mean zero, standard deviation 0.1), to take into account the fact that resistance is never either total or completely absent. Finally, the regions of origin were plotted using colours, using the matplotlib library.*

Reviewer #2: Are the objectives of the study clearly articulated with a clear testable hypothesis stated?

**While this study does not have a specific hypothesis stated, it aims to address several questions regarding the distribution of L4 and L5 sublineages in Nigeria compared to neighboring countries, such as Ghana and Cameroon, and to infer information about the origin of L4.6.2. It also seeks to investigate the association between specific sublineages of L4 and L5 and the emergence and transmission of antimicrobial resistance.**

-Is the study design appropriate to address the stated objectives?
**Yes**


-Is the population clearly described and appropriate for the hypothesis being tested?
**The authors utilized genomic data from Nigeria (n=116), Cameroon (n=202), and Ghana (n=175), along with an additional analysis of 24 strains sequenced from Northern Nigeria.**


-Is the sample size sufficient to ensure adequate power to address the hypothesis being tested?
**The authors acknowledge the importance of analyzing tuberculosis strains in Northern Nigeria; however, it is noted that the number of strains used in the analysis is limited and may not be considered sufficient**.

*The authors recognize the importance of analyzing tuberculosis strains in Northern Nigeria; however, they acknowledge that the limited number of strains used for the analysis may not be sufficient.*

-Were correct statistical analysis used to support conclusions?
**Yes**

-Are there concerns about ethical or regulatory requirements being met?
**No**

--------------------

<b>Results</b></br></br>
-Does the analysis presented match the analysis plan?</br>
-Are the results clearly and completely presented?</br>
-Are the figures (Tables, Images) of sufficient quality for clarity?</br></br>

R**eviewer #1: The supplemental data must be presented with more context**.

*Answer : we added more information (legend improved) and checked all references relative to these Supplementary data and improved the appropriateness of citation, hence, we are now providing more contextual information to the supplementary data*

**The "supercomputer analysis" is too much a black box and must be further explained.**

*Answer: we now include a reference (DOI) to a BioRxiv preprint that describes in detail the principle of the "TB-Annotator"*

*Senelle G, Guyeux C, Refrégier G, Sola C. TB-Annotator: a scalable web application that allows in-depth analysis of very large datasets of publicly available Mycobacterium tuberculosis complex genomes. bioRxiv 2023.06.12.526393; doi: https://doi.org/10.1101/2023.06.12.526393*


Reviewer #2: -Does the analysis presented match the analysis plan?
**Yes**

-Are the results clearly and completely presented?

**The numbers representing resistance rates in Figure 6 lack clarity regarding the underlying population.**

*Answer: The figure 1 and 6 were replaced by the Figure below*

*the legend is:*

*Number of individuals per state in the cohort of 165 patients. The percentage corresponds to the number of isoniazid-resistant patients.*

-Are the figures (Tables, Images) of sufficient quality for clarity?

**The authors should re-draw the molecular clock phylogenetic analysis in Supplementary Material 5 so that the strain name can be read.**

*Answer: We believe that the reviewer meant Supplementary Material 6 (dated phylogeny of M. bovis phylogenetical network) and not Supplementary Material 5 which is a star-shaped phylogenetical network showing a particular Nigerian branch. Supplementary Material 6 was redrawn (now designated as Supplementary Material 4), and the strain name can be read now, done*


--------------------

<b>Conclusions</b></br></br>
-Are the conclusions supported by the data presented?</br>
-Are the limitations of analysis clearly described?</br>
-Do the authors discuss how these data can be helpful to advance our understanding of the topic under study?</br>
-Is public health relevance addressed?</br></br>

Reviewer #1: **The conclusion the authors suggest, is not clearly supported by the data and the link between the data and the result must be stated more clearly.**

*Answer: we deleted any overstatement that is not supported by the data.*

**Limitations of analysis and possible alternative scenarios should be expanded upon.**

Answer: concerning the origin of L4.6.2.2, we introduced the following sentence :

*An alternate scenario of endemicity of L4.6.2 to Western-central Africa (Cameroun-Nigeria) is also possible.*

**Looking at table 3 I disagree, that "This result confirms in Northern Nigeria the previous distribution maps obtained 5 years ago by spoligotyping directly on sputum samples [6] and definitively demonstrates that L4.6.2.2 is responsible of RIF resistance and MDRTB progression in Nigeria". This should be further discussed.**

*Answer: this sentence was deleted*

Reviewer #2: -Are the conclusions supported by the data presented?

**The results of the molecular clock phylogenetic analysis are interpreted and discussed in relation to the historical background, but the evidence is not explained at all. The authors should provide a thorough explanation, including references, to support their interpretation and discussion.**

*Answer: we now added historical references which could support our scenario:*

1.	Austen RA. *Trans-Saharan History in World History*. NYC: Oxford University Press; 2010.

2.	Simpson A. Oral tradition and slave trade in Nigeria, Ghana and Benin. Paris: UNESCO, 2004.

-Are the limitations of analysis clearly described?
**Yes**

-Do the authors discuss how these data can be helpful to advance our understanding of the topic under study?

**In this paper, while the focus is primarily on presenting the analysis and findings, it also emphasizes the need for further accumulation of genomic data on tuberculosis (TB) in West Africa. By highlighting the genetic diversity, transmission patterns, and antimicrobial resistance profiles observed in the studied population, the paper implicitly suggests that** **expanding the genomic data repository in this region would greatly enhance our understanding of TB epidemiology and evolution**.

-Is public health relevance addressed?
No

--------------------

<b>Editorial and Data Presentation Modifications?</b></br><br/>
Use  this section for editorial suggestions as well as relatively minor modifications of existing data that would enhance clarity. If the only modifications needed are minor and/or editorial, you may wish to recommend “Minor Revision” or “Accept”.

Reviewer #1:

**Supp Mat 2, 4, 5! and 6 must be presented in a clearer way and explained further**.

*One supplementary file was suppressed, the other are better introduced and their legend was improved*

**Table 3: remove decimal places for clarity and overview**.

*done*

Reviewer #2:

**＃１The authors should clearly state how they ensure data quality with respect to data downloaded from NCBI**.

*Answer :*

*We ensure the quality of the data we download from NCBI by following strict criteria, as explained in our now referenced TB annotator paper. As the article on our analysis platform has now been referenced, we did not feel it necessary to add further details in this article, so as not to lose the reader. For the reviewer's information, here is a closer look at what we do.*

*Sequences are then downloaded from Sequence Read Archive with fastq-dump, as paired-end or single-end FASTQ files. These files contain sequencing reads with the sequence and a quality score for each base, the PHRED score. These scores are used in the reads pre-processing and mapping steps to assess the reads quality. Genome coverage quality is verified based on the number of reads in the FASTQ file using SeqKit. Fastp is then used for quality control on the reads and to pre-process them. In the pre-processing part, the adapters used in library preparation process during sequencing are trimmed. Low quality bases at ends of reads are cut and reads with too many low quality bases are removed. For paired-end reads that are overlapping, they are corrected based on quality score if they are not a perfect reverse-complement. Some advanced corrections are also performed like polyG trimming and UMI preprocessing.*

*Following the read quality control and pre-processing, a report is outputted in html and json formats. The report contains informations and statistics about the different parts of the reads filtered, base contents, and duplication rates. This report is used to evaluate the sequencing process, and detect problems like contaminated reads, base content biases and over-represented sequences.*

*TB-annotator: a scalable web application that allows in-depth analysis of very large sets of publicly available Mycobacterium tuberculosis complex genomes. Gaetan Senelle, Christophe Guyeux, Guislaine Refrégier, and Christophe Sola. bioRxiv 2023.06.12.526393; doi: https://doi.org/10.1101/2023.06.12.526393*

**＃２The authors have only 24 newly read sequences in Northern Nigeria. I recommend sequencing of the 165 specimens for which drug susceptibility data were obtained.**

*Answer: we agree with the reviewer that the sequencing of all DR isolates would have been better. The PhD project was initially focusing on L5. The recruitment of L5 samples was low as compared to our first estimates, hence we also characterized L4 MDR-TB isolates by whole-genome sequencing. The sample was quite low because the primary dedicated budget allocated would not have allowed to sequence all MDR-TB isolates.*

--------------------

<b>Summary and General Comments</b></br></br>
Use  this section to provide overall comments, discuss strengths/weaknesses of the study, novelty, significance, general execution and scholarship. You may also include additional comments for the author, including concerns about dual publication, research ethics, or publication ethics. If requesting major revision, please articulate the new experiments that are needed.

Reviewer #1: **The headline should be adapted to represent better that mostly the lineages in Nigeria and the phylogenetics are focused on**.

*Answer : the title was adapted accordingly, following the reviewer suggestions*

***Mycobacterium tuberculosis* complex drug-resistance, phylogenetics, and evolution in Nigeria: comparison with Ghana and Cameroon**

Reviewer #2: (No Response)

--------------------

PLOS authors have the option to publish the peer review history of their article (<a href="https://journals.plos.org/plosntds/s/editorial-and-peer-review-process#loc-peer-review-history" target="_blank">what does this mean?</a>). If published, this will include your full peer review and any attached files.<br><br>

If you choose “no”, your identity will remain anonymous but your review may still be made public.<br><br>

<b>Do you want your identity to be public for this peer review?</b> For information about this choice, including consent withdrawal, please see our <a href="https://www.plos.org/privacy-policy" target="_blank">Privacy Policy</a>.

Reviewer #1: Yes: **Oliver David Komm**

Reviewer #2: **No**


Figure Files:
While revising your submission, please upload your figure files to the Preflight Analysis and Conversion Engine (PACE) digital diagnostic tool, https://pacev2.apexcovantage.com. PACE helps ensure that figures meet PLOS requirements. To use PACE, you must first register as a user. Then, login and navigate to the UPLOAD tab, where you will find detailed instructions on how to use the tool. If you encounter any issues or have any questions when using PACE, please email us at figures@plos.org.
 
Data Requirements:
Please note that, as a condition of publication, PLOS' data policy requires that you make available all data used to draw the conclusions outlined in your manuscript. Data must be deposited in an appropriate repository, included within the body of the manuscript, or uploaded as supporting information. This includes all numerical values that were used to generate graphs, histograms etc.. For an example see here: http://www.plosbiology.org/article/info%3Adoi%2F10.1371%2Fjournal.pbio.1001908#s5.
 
Reproducibility:
To enhance the reproducibility of your results, we recommend that you deposit your laboratory protocols in protocols.io, where a protocol can be assigned its own identifier (DOI) such that it can be cited independently in the future. Additionally, PLOS ONE offers an option to publish peer-reviewed clinical study protocols. Read more information on sharing protocols at https://plos.org/protocols?utm_medium=editorial-email&utm_source=authorletters&utm_campaign=protocols

References
Please review your reference list to ensure that it is complete and correct. If you have cited papers that have been retracted, please include the rationale for doing so in the manuscript text, or remove these references and replace them with relevant current references. Any changes to the reference list should be mentioned in the rebuttal letter that accompanies your revised manuscript. If you need to cite a retracted article, indicate the article's retracted status in the References list and also include a citation and full reference for the retraction notice.

__________________________________________________
In compliance with data protection regulations, you may request that we remove your personal registration details at any time.  (Use the following URL: https://www.editorialmanager.com/pntd/login.asp?a=r). Please contact the publication office if you have any questions.
